# Identification of Rickettsiae, Uganda and Djibouti

**DOI:** 10.3201/eid1310.070078

**Published:** 2007-10

**Authors:** Cristina Socolovschi, Kotaro Matsumoto, Jean-Lou Marie, Bernard Davoust, Didier Raoult, Philippe Parola

**Affiliations:** * Faculté de Médecine Marseille, Marseille, France; † Service de Santé des Armées, Marseille, France; ‡ Direction Régionale du Service de Santé des Armées, Toulon, France

**Keywords:** Ticks, Rickettsia, Uganda, Djibouti, Africa, letter

**To the Editor:** Tickborne rickettsioses are caused by obligate intracellular gram-negative bacteria that belong to the spotted fever group of the genus *Rickettsia*. These zoonoses share characteristic clinical features, including fever, headache, rash, and sometimes eschar formation at the site of the bite (*1*). Although rickettsioses are important emerging vectorborne infections of humans worldwide, little is known about rickettsioses in sub-Saharan Africa (*1*,*2*).

In 2002, 94 ticks were collected in Djibouti: 5 *Amblyomma lepidum*, 1 *A. variegatum*, 5 *Hyalomma marginatum rufipes,* 40 *Rhipicephalus* *pulchellus,* and 10 *Rh. evertsi evertsi* from cattle that had just arrived from Ethiopia; 30 *H. dromedarii* from dromedaries; and 3 *Rh.* *sanguineus* group ticks from cheetahs. In 2003, 57 ticks were collected from dogs in Kampala, Uganda: 1 *A. variegatum,* 9 *Haemaphysalis punctaleachi,* 28 *Rh. praetextatus,* and 19 *Rh. sanguineus*. All ticks were partially or fully engorged adults. This convenience sample of ticks was obtained as part of other ongoing studies.

Ticks were identified by using taxonomic keys (*3*) and kept in 70% ethanol before being tested. DNA of each tick was extracted, and rickettsial DNA was detected by PCR that used primers Rp.877p and Rp.1258r, which amplify a 396-bp fragment of the citrate synthase gene (*gltA*) of rickettsia, as described (*4*). Rickettsia-positive samples were tested by a second PCR that used Rr.190.70p and Rr.190.701n primers, which amplify a 629–632 bp fragment of *ompA* gene (*4*). Controls (2 negative [DNA extracted from noninfected laboratory ticks and distilled water] and 1 positive [*R. montanensis* DNA]) were included in each test. The sequences of PCR products were obtained and compared with those available in GenBank (*4*).

One specimen of *Ha. punctaleachi* from Uganda and 1 *A. lepidum* from Djibouti, as well as positive controls, were positive according to PCR using both primer pairs. No signal was obtained from negative controls. The sequence of a 474-bp fragment of o*mpA* obtained from *Ha. punctaleachi* showed 99.8% (473/474) similarity with *R. conorii* (GenBank accession no. AY346453); those of a 340-bp segment of *gltA* showed 100% similarity with that of *R. conorii* (AE008677). The sequences of a 517-bp segment of *omp*A and a 341-bp segment of *glt*A amplified from *A. lepidum* showed 100% similarity to the corresponding sequences of *R. africae* (U83436 and U59733, respectively)*.*

To our knowledge, this is the first detection of *R. conorii*, the agent of Mediterranean spotted fever, in Uganda. Although the main vector of this rickettsia is *Rh. sanguineus*, the few ticks of this species we tested were negative (*1*). It is also the first detection of *R. conorii* in *Ha. punctaleachi*, although it has been detected in the closely related *Ha. leachi* in Zimbabwe (*5*). *Ha. punctaleachi* prefers warm and humid conditions but can exist wherever rodent hosts for its immature stages and canine hosts for its adult stages are present (*6*). Adults are found throughout the year; peak numbers occur either from winter to early summer or from spring to late summer (*7*). Although the detection of *R. conorii* in *Ha. punctaleachi* does not mean that this tick is an efficient vector (*8*), clinicians in Uganda should be aware of the presence of Mediterranean spotted fever in their country.

This is also, to our knowledge, the first detection of *R. africae*, the agent of African tick bite fever, in Djibouti. *R. africae* was also detected in 1 *A. lepidum* collected in Sudan (*4*), but it is more frequently detected in *A. variegatum* and *A. hebraeum* with high infection rates throughout sub-Saharan Africa (*7*). *A. lepidum*, which coexists with *A. variegatum* in limited locations, is chiefly a cattle parasite. It will also attach to smaller domestic animals and a few wild herbivores, but it attacks humans less frequently than *A. variegatum* or *A. hebraeum*. *A. lepidum* occurs in a variety of climatic regions but most commonly inhabits semiarid regions in eastern Africa. The cattle in our study had been imported from Ethiopia, and the ticks may have infested these animals before their arrival in Djibouti. Indeed, in 1973 Burgdorfer obtained an isolate from *A. variegatum* in Ethiopia, which was thereafter shown to be indistinguishable from the rickettsia described as *R. africae* (*7*,*9*). Again, clinicians should be aware of the presence of *R. africae* in Djibouti and that it could affect their patients, both local and international, including French and American soldiers based in this country (*10*).

Because we did not do systematic sampling, our results cannot address the prevalence and distribution of *R. conorii* and *R. africae* in Uganda and Djibouti, respectively. However, healthcare workers who treat persons who live in or have traveled to these countries should be alert for spotted fever group rickettsial infections in their patients (*1*).
